# The Evolving Association of Social Determinants of Health and Vaccination Coverage Among Older Adults: A Neighborhood-Level Analysis of COVID-19

**DOI:** 10.3390/vaccines14050387

**Published:** 2026-04-26

**Authors:** Seyed M. Karimi, Brendan Sullivan, Venetia Aranha, Mana Moghadami, Md Yasin Ali Parh, Shaminul H. Shakib, Hamid Zarei, Trey Allen, Yuting Chen, Taylor Ingram, Angela Graham

**Affiliations:** 1 Department of Health Management and Systems Sciences, School of Public Health and Information Sciences, University of Louisville, Louisville, KY 40202, USA; mana.moghadami@louisville.edu (M.M.); hamid.zarei@louisville.edu (H.Z.); 2 Division of Population Health, Louisville Metro Department of Public Health and Wellness, Louisville Metro Government, Louisville, KY 40202, USA; trey.allen@louisvilleky.gov (T.A.); yuting.chen@louisvilleky.gov (Y.C.); angela.graham@louisvilleky.gov (A.G.); 3 Department of Bioinformatics and Biostatistics, School of Public Health and Information Sciences, University of Louisville, Louisville, KY 40202, USA; brendan.sullivan.1@louisville.edu; 4 Department of Epidemiology and Population Health, School of Public Health and Information Sciences, University of Louisville, Louisville, KY 40202, USA; venetia.aranha@louisville.edu; 5 Communicable Disease Surveillance and Education, Louisville Metro Department of Public Health and Wellness, Louisville Metro Government, Louisville, KY 40202, USA; 6 Department of Biostatistics, University of Michigan, Ann Arbor, MI 48109, USA; yasinp@umich.edu; 7 Department of Public Health, College of Health Sciences, Sam Houston State University, Huntsville, TX 77340, USA; shs017@shsu.edu; 8 Down Syndrome of Louisville, Louisville, KY 40291, USA; taylori@dsoflou.org

**Keywords:** COVID-19 vaccination, older adults, vaccine equity, disability, poverty rate, social determinants of health, spatial analysis, Jefferson County, Kentucky

## Abstract

**Background:** Older adults (aged 65 and older) faced a disproportionate burden of mortality during the COVID-19 pandemic, yet substantial geographical and sociodemographic disparities in vaccine uptake persisted within this vulnerable population. **Objective:** To examine the temporal dynamics of COVID-19 vaccination rates among older adults and investigate the association between vaccination uptake and neighborhood-level social determinants of health (SDOHs), including disability and poverty. **Methods:** COVID-19 vaccination data for older adult residents in Jefferson County, Kentucky, were obtained from the Kentucky Immunization Registry (KYIR). ZIP-code-level vaccination rates were calculated at three time points: 28 February 2021 (Q1), 31 May 2021 (Q2), and 31 May 2022 (Q6). The rates were linked to 2021 American Community Survey (ACS) ZIP code-level estimates of disability, poverty, and household composition. Two-dose COVID-19 vaccination rates stratified by race, ethnicity, and geographic region were used as outcome measures. Pearson correlation coefficients, bivariate, and multivariate linear models were used to estimate the association between COVID-19 vaccination rates and the SDOHs at the ZIP code level. **Results:** Among the estimated 139,222 older adults, overall two-dose vaccination rates rose from 22.4% in Q1 to 77.5% by Q6. Significant regional disparities were observed early in the campaign, with Q1 rates ranging from 12.6% in the Southwest to 35.4% in the Inner East county regions. Bivariate analyses showed ZIP-code-level disability and poverty rates were negatively associated with ZIP-code-level vaccination uptake in Q1 (disability slope: −0.38; 95% CI, −0.63 to −0.13; poverty slope: −0.36; 95% CI, −0.65 to −0.07). By Q6, the negative association between disability and vaccination had weakened significantly and was no longer statistically significant, while the negative association between poverty rate and vaccination rate remained persistent across all time points. **Conclusions:** The disability-associated gaps in older adults’ vaccination rates were dynamic and narrowed over time, whereas the poverty-associated gaps remained persistent and static. The low uptake observed among Black and Hispanic older adults in historically underserved areas suggests that understanding the specific factors that most negatively associate with vaccination rates in these populations, such as specific disabilities, may mitigate structural barriers. Future public health interventions should prioritize socioeconomically disadvantaged neighborhoods and account for the evolving association of functional impairments and healthcare access.

## 1. Introduction

Vaccination is key to preventing infectious diseases, but it is particularly important for older adults (65 years and older) due to the higher prevalence of underlying conditions and a declining immune system [1,2,3], as, for example, approximately 90% of influenza deaths occur in older adults in the United States (U.S.) [4]. Moreover, an alarmingly high rate, 75%, of COVID-19-associated mortalities were in older adults in the U.S., despite only representing 12% of COVID-19 cases [5]. Nonetheless, vaccination rates among older adults have remained below target levels [6,7].

The high impact of diseases such as influenza and COVID-19 on older adults underscores the need to understand the factors driving suboptimal vaccination rates in this population. Based on the Anderson healthcare utilization model, such factors can be categorized into three groups: (1) predisposing factors: individual and structural factors, such as education and demographic characteristics, that influence older adults’ predisposition to uptake vaccines; (2) enabling factors: individual and community level resources, such as health insurance coverage, accessibility of healthcare services, and income, that enable or impede their utilization of care; and (3) need factors: older adults’ healthcare needs influenced by the presence of chronic diseases and disability [8].

Predisposing factors such as education, race, ethnicity, sex, and marriage status are strong correlates of influenza, pneumococcal, Tdap, and herpes zoster vaccination among older adults in the U.S., such that lower education levels, being in a racial or ethnic minority group, being male, and being unmarried are associated with lower uptake of these vaccines [9,10,11]. The influence of enabling factors on older adults’ vaccination rates is also strong. For example, having either private or public health insurance is associated with a higher likelihood of receiving a COVID-19 vaccine [5], and Medicare Advantage plans are associated with a greater likelihood of receiving an influenza vaccine than the traditional Medicare plan [10]. Higher income and access to a trusted physician have also been found to increase vaccination rates among older adults [10,11].

The evidence on the association of vaccination and a key need factor, disability, among older adults is scarce and inconclusive. One study of adults aged 18 or older found that disability was associated with a reduced likelihood of influenza vaccination [12]. Another study showed that disability was associated with increased odds of receiving the influenza vaccine among U.S. older adults, adjusting for a set of predisposing and enabling factors [9]. Also, a study of older adults in Florida found a positive association between disability and influenza and pneumococcal vaccination [13]. Evidence on the association between COVID-19 vaccination and disability, however, is more consistent: disability was associated with a lower likelihood of COVID-19 vaccination among older adults in multiple studies [14,15,16].

Another key determinant of healthcare utilization is geography, which serves as both a predisposing and enabling factor: the neighborhood environment can predispose older adults to specific health beliefs and attitudes towards vaccination, and it can also determine proximity to healthcare facilities. Geographical differences, even at small scales such as the neighborhood level, yield different health outcomes, including infection rates. In New York City, socially disadvantaged neighborhoods, where residents were less able to socially isolate and more likely to use the subway, had higher COVID-19 infection rates [17]. Also, a population-based study of Massachusetts ZIP codes revealed wide geographic variation in COVID-19 vaccination and booster coverage, with significant inequities driven by neighborhood-level differences in education, median household income, and the concentration of essential workers [18].

There is limited knowledge of vaccination rates among older adults in the U.S., particularly at the intersection of race, ethnicity, disability, income, and living conditions within geographic units as granular as ZIP codes. In addition, the temporal dynamics of the relationship between vaccination and these predisposing, enabling, and need factors are largely unknown. This study aims to address these knowledge gaps by examining COVID-19 vaccination data from the City of Louisville/Jefferson County, Kentucky, USA (2024 estimated population = 793,881) [19], immunization registry. By conducting a population-level analysis within a specific county, this study is substantially less affected by significant differences in vaccination policy, as well as cultural and environmental heterogeneities, which often have a major impact in larger-scale studies.

## 2. Materials and Methods

**Figure A1:**
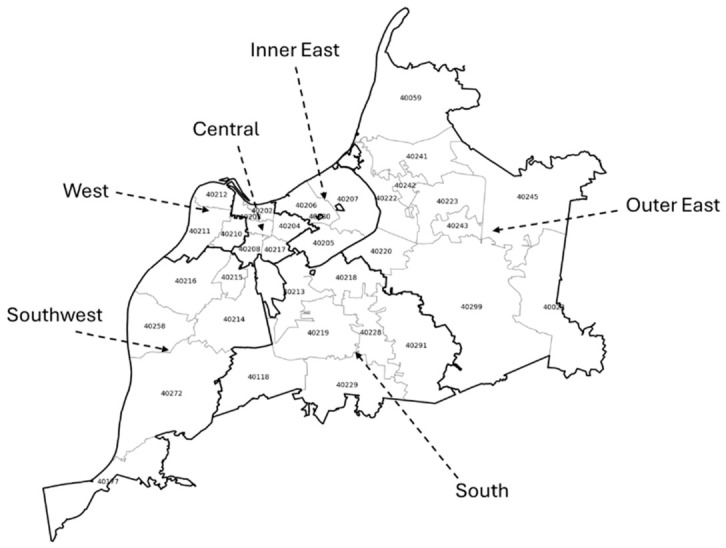
Jefferson County, Kentucky, ZIP codes and regions map.

The dataset for this study was obtained from the Kentucky Immunization Registry (KYIR) by the Louisville Metro Department of Public Health and Wellness (LMPHW). The provided data included population-level COVID-19 vaccination information for Jefferson County, Kentucky, residents (vaccine type and vaccination dates), along with their demographic characteristics (sex, age in single years, race, and ethnicity), and residential ZIP codes (which are equivalent to postal codes in most countries). The two-dose COVID-19 vaccination rates at the end of Quarter 1 (28 February 2021), Quarter 2 (31 May 2021), and Quarter 6 (31 May 2022) of the COVID-19 vaccination campaign in the county were calculated by race and ethnicity in the county’s ZIP codes. The rates were also calculated in six groups of ZIP codes, named county regions, formed based on the county’s main market areas, which are regularly used in disease surveillance by the LMPHW. The formed county regions did not include ZIP codes assigned to P.O. Boxes, the city’s international airport, or areas with a population below 1000. The county regions were West, Southwest, South, Central, Inner East, and Outer East (Figure A1).

The numerator for the calculated vaccination rates was the number of older adults who received at least two doses of a COVID-19 vaccine in each racial and ethnic category; the denominator was the 2021 population in each category. The numerators were extracted from the KYIR data; the denominators were estimated by the geometric extrapolation of single-year ZIP code-level population data by age, race, and ethnicity from the 2010 and 2020 Censuses.

The analyzed racial groups were Asian, Black, Multiracial, Some Other Races, and White. American Indian/Alaska Natives and Native Hawaiian/Other Pacific Islanders were excluded due to very small populations in the county [19]. Ethnicity groups included Hispanic and non-Hispanic.

Correlations between ZIP code-level vaccination rate and a set of social determinants of health (SDOHs) for older adults (namely, the disability rate, the poverty rate, the share of households with an older adult, and the rate of older adult householders living alone) were calculated using the Pearson product-moment correlation coefficient. The SDOH measures were obtained from the U.S. Census Bureau’s 2021 American Community Survey (https://data.census.gov/advanced (accessed on 7 November 2025)). No other older adult SDOH at the ZIP code level was identified in the U.S. Census Bureau Data Portal for examination. The correlation coefficients were calculated at the end of the first, second, and sixth quarters of the COVID-19 vaccination to assess the temporal dynamic of the relationship between vaccination rate and an SDOH.

Since Pearson correlation coefficients do not provide a determination of statistical significance, the relationship between vaccination rates and each SDOH at the ZIP code level was estimated using a bivariate linear model. The slope coefficient of the model provides an estimate of the correlation between vaccination rate and the SDOH. The estimated models also provide 95% confidence intervals for the coefficients. Such linear models were estimated at the end of the first, second, and sixth quarters of the COVID-19 vaccination. In addition, a correlation matrix of the SDOHs was generated, and multivariate linear models were estimated to assess the overlapping associations of the SDOHs with the COVID-19 vaccination rate.

Vaccination rates and SDOHs information at the county region level are provided in the main text tables; ZIP code-level rates and information are provided in the tables of Appendix A. Also, the results of multivariate analyses are provided in the tables of Appendix A. For data analysis, table creation, and map generation, Stata v.18, Microsoft Excel, and Python 3.11 were used, respectively.

## 3. Results

In 2021, the county’s estimated population of older adults was 139,222 (Table 1, Table A1 and Table A2). Their composition was 79.8% White, 15.0% Black, 3.2% Multiracial, 1.7% Asian, and 0.8% Some Other Race. Also, 2.2% of the older adult population was Hispanic, versus a 98.0% non-Hispanic older adult population (the 0.5% discrepancy in racial population estimation and the 0.2% discrepancy in ethnic population estimation is due to population estimation error). The Outer East region had the highest number of older adults in the county (48,630; 34.9%), followed by the South (30,846; 22.2%) and Southwest (27,227; 19.6%). The Inner East (15,686; 11.3%) and Central (8193; 5.9%) had comparatively smaller shares of the older adult population, while the West region (7888; 6.7%) was the least populous region for older adults. The county’s most populous ZIP codes for older adults (40299 and 40291) were in the Outer East and South regions, respectively.

The majority of White older adults lived in the Outer East region (42,819), where they comprised 88.1% of the older adult population (Table 1). The county region with the fewest White older adults was the West (1268; 16.1%). On the other hand, there were 6388 Black older adults in the West region, and they composed 81.0% of the older adult population in this region. The fewest Black older adults lived in the Inner East region (575; 3.7%). Most Asian older adults lived in the Outer East (1058; 2.2%), and they were least prevalent in the West (15; 0.2%). The highest number of Multiracial older adults was in the South (1333; 4.3%), and the lowest number was in the West (176; 2.2%). Most of the older adults of Other Races lived in the South (478; 1.5%), and the fewest lived in the West (40; 0.5%). The majority of Hispanic older adults lived in the South region (1140; 3.7%), and the fewest lived in the West (86; 1.1%).

Substantial regional and ZIP-code-level disparities in the two-dose COVID-19 vaccination rate among older adults were present, though they diminished over time (Table 2, Table A3, Table A4 and Table A5). At the end of the first quarter of the vaccination campaign (28 February 2021), the rates of vaccination among older adults were the highest in the Inner East and Outer East regions at 35.4% and 29.8%, respectively, and the lowest in the West and Southwest regions at 12.8% and 12.6%, respectively (Table 2). Between Quarter 1 and Quarter 2 of the vaccination campaign, vaccination rates grew the most in the South, Southwest, and West regions―increasing by 52%, 51.8%, and 50.1% on 31 May 2022, respectively. Still, vaccination rates in these regions remained remarkably lower than those in the Inner East and Outer East regions: the Inner East and Outer East had vaccination rates of 78% and 75.7%, respectively, at the end of the second quarter of vaccination (31 May 2021), while the highest vaccination rate during this quarter among the South, Southwest, and West regions was 67.4.%, in the South. By Quarter 6, the West and Southwest regions both reached 74.1%, while the Inner East and Outer East reached 83.3% and 80.0%, respectively. Hence, the gap persisted, but it shrank over time.

**Figure 1:**
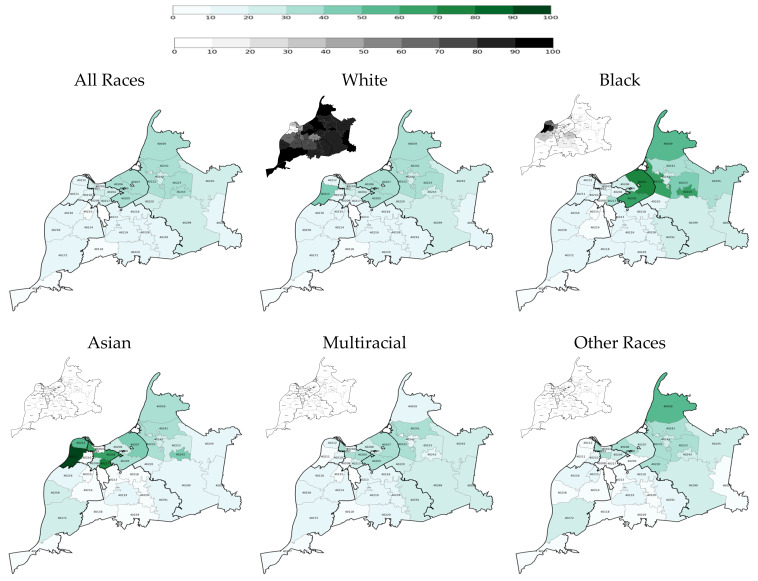

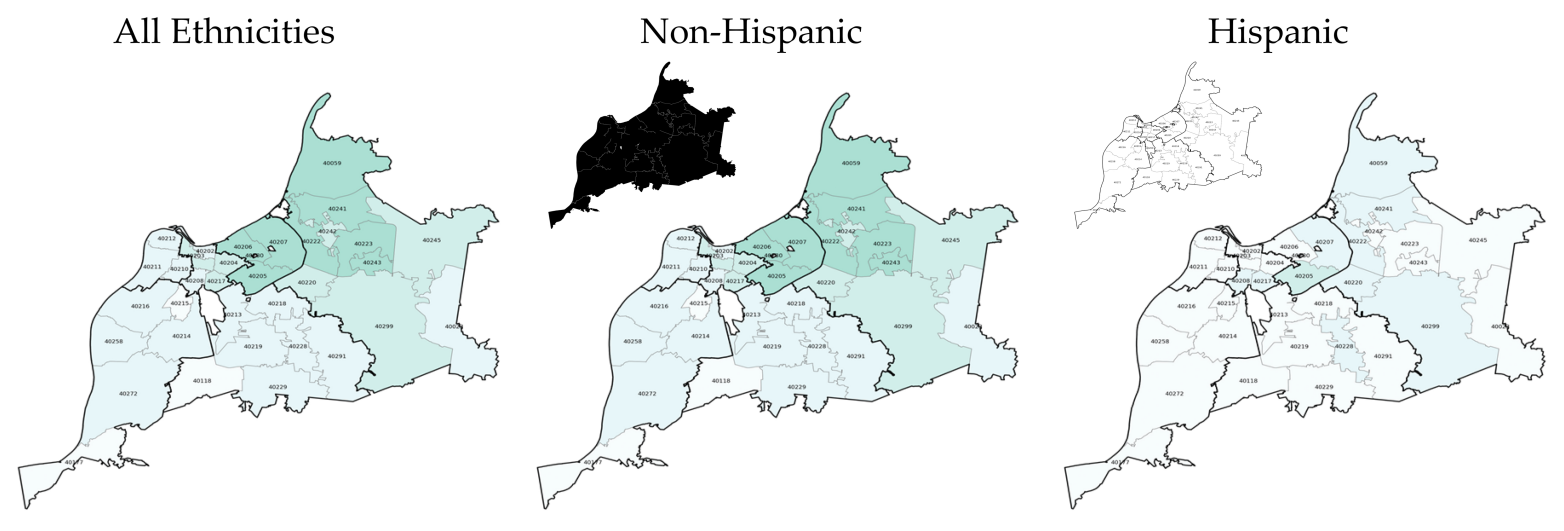
Two-dose COVID-19 vaccination rate among older adults at the end of the first quarter of the vaccination campaign on 28 February 2021 by race, ethnicity, and ZIP code (population density maps located at the top left corners).

**Figure 2:**
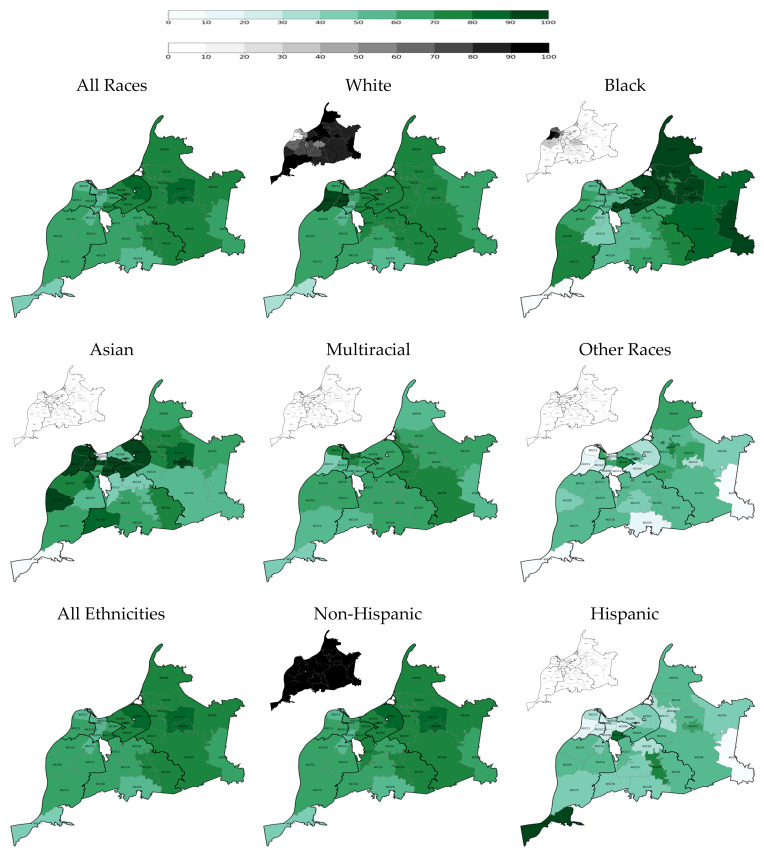
Two-dose COVID-19 vaccination rate among older adults at the end of the second quarter of the vaccination campaign on 31 May 2021 by race, ethnicity, and ZIP code (population density maps located at the top left corners).

**Figure 3:**
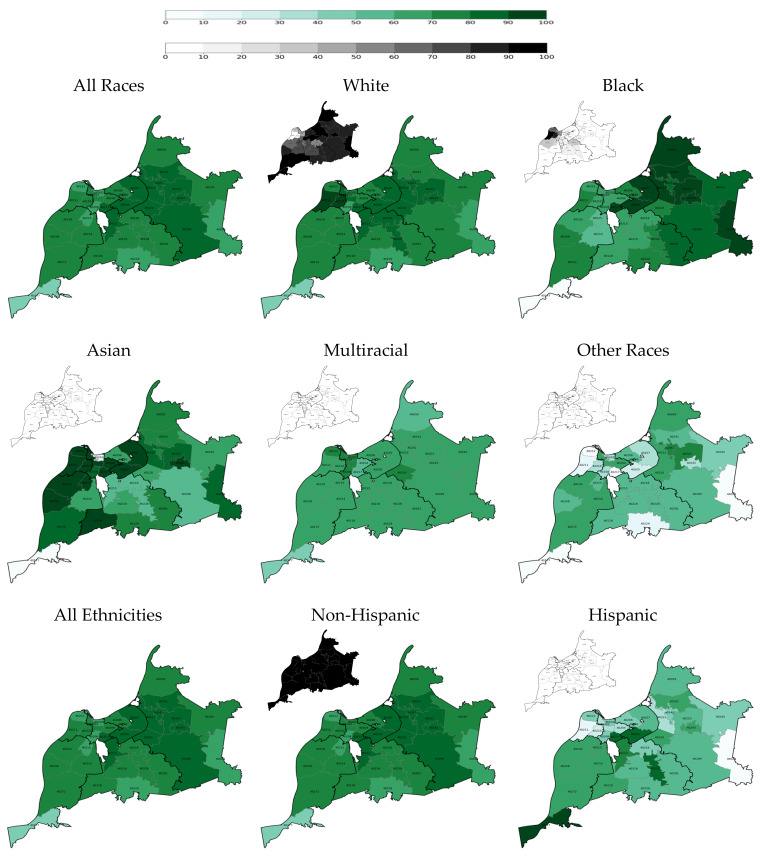
Two-dose COVID-19 vaccination rate among older adults at the end of the sixth quarter of the vaccination campaign on 31 May 2022 by race, ethnicity, and ZIP code (population density maps located at the top left corners).

Among racial groups, White older adult residents had the highest two-dose vaccination rate at each time point: 23.7% on 28 February 2021 and 71.6% on 31 May 2021, and 78.3% on 31 May 2022 (Table 2 and Figure 1, Figure 2 and Figure 3). Throughout the vaccination campaign, vaccination rates among White residents varied widely across the county, with the Inner East and Outer East regions having the highest rates, and the Southwest and South regions having the lowest. On the other hand, Black older adult residents had a consistently low two-dose vaccination rate at the end of each quarter: 16.5% on 28 February 2021 (the lowest vaccination rate among racial groups at the end of quarter 1), 65.5% on 31 May 2022, and 73.3% on 31 May 2022. The vaccination rate among Black residents also varied widely across the county, with the Inner East and Outer East regions again having the highest rates, and the West and Southwest regions having the lowest rates during the vaccination campaign. Additionally, the overall White and Black vaccination rates increased at similar rates: the overall Black vaccination rate increased by 49 percentage points between Quarter 1 and Quarter 2 and by 7.8 percentage points between Quarter 2 and Quarter 6; the overall White vaccination rate increased by 47.9 percentage points between Quarter 1 and Quarter 2 and by 6.7 percentage points between Quarter 2 and Quarter 6. As such, the overall White-Black gap in COVID-19 immunization remained persistent.

Furthermore, Some Other Races and Hispanic older adults had persistently low vaccination rates, even in Quarter 6, particularly in the West Region (Table 2 and Figure 1, Figure 2 and Figure 3). However, it is pertinent to note that there were only 40 older adults in the Some Other Races category in the West region, and only 86 Hispanic older adults in this county region. Nonetheless, it is noteworthy that the Hispanic overall vaccination rate was the lowest until Quarter 6, when the overall vaccination rate among members of Some Other Races reached its lowest point.

While the West, Southwest, and South regions had the largest overall increases in vaccination rates, by the end of Quarter 6, the overall vaccination rates remained highest in the Central, Inner East, and Outer East regions (Table 2).

By Quarter 6, while the lowest regional vaccination rates (in the West and Southwest) were both 74.1%, the vaccination rate among Hispanic residents was 28.3%, and the vaccination rate among those of Some Other Races was 21.0% within the West region (Table 2).

Finally, within the Multiracial category, some vaccination percentages decreased from Quarter 2 to Quarter 6 (Table 2). This is due in part to point-estimation error caused by population point estimates that corresponded to the lower end of the U.S. Census Bureau’s 2021 confidence intervals.

Of the four SDOH variables included in the analysis, the most prevalent overall was the percentage of older adult householders living alone (Table 3 and Table A5). The percentage of older adults living with a disability was highest in the West region (51.4%) and lowest in the Inner East region (26.3%). In the Central region, 26.0% of older adults were living below the poverty line, the highest among the county regions; the Outer East had the lowest older adult poverty rate at 5.4%. The region with the highest percentage of households with an older adult was the Outer East (33.1%), and the lowest was the Central region (21.4%). The Central region had the highest percentage of older adult householders living alone (67.9%), while the Outer East had the lowest (44.0%).

Bivariate analyses showed a statistically significant relationship between disability and vaccination rates at the ZIP code level in Quarter 1 (Table 4). Additionally, when comparing regression slopes within each SDOH variable across vaccination quarters, hypothesis testing showed that only the slope of disability rate differed significantly between Quarter 1 and Quarter 6. As disability rate increased, the vaccination rate decreased in Quarter 1. In Quarter 6, this relationship between the disability rate and vaccination rate persisted, but the vaccination rate decreased much less than previously. Poverty rate and the share of households with a 65+ person remained statistically significant correlates of vaccination rate for all 3 Quarters. Still, the rate of 65-plus householders living alone was never statistically significantly associated with vaccination rate. Similar results were found when the top and bottom 10% of SDOH were excluded (Table A7).

Multivariate analyses were conducted after accounting for findings from the SDOHs’ correlation matrix, indicating an approximately 78% correlation between the poverty rate and the rate of 65+ householders living alone (Table A8). Consequently, the rate of 65+ householders living alone was excluded from the multivariate analyses due to its strong correlation with the poverty rate and its lack of bivariate correlation with the vaccination rate. A different set of multivariate linear regressions was estimated for each of the remaining three SDOHs by adding other SDOHs to the corresponding bivariate regression to identify overlapping determinants of the vaccination rate across SDOHs (Table A9, Table A10 and Table A11). The bivariate association between older adults’ vaccination rate and the rates of disability and households with older adults was largely preserved after adjusting for other SDOHs (Table A9 and Table A11). Similar consistency was not observed in the association between the older adults’ vaccination rate and the poverty rate, although it approached statistical significance in Q2 and Q6 (Table A10).

## 4. Discussion

### 4.1. Older Adults’ COVID-19 Vaccination Rate over Time

Most older adults in Jefferson County, Kentucky, were fully vaccinated in the first two quarters of the COVID-19 vaccination campaign, resulting in vaccination rates of 22.4% in Quarter 1 and 70.5% in Quarter 2 (Table 2). The main reason for the sharp increase in uptake was the prioritization of vaccination for older adults. As of 22 February 2021, 41 states and the District of Columbia had expanded COVID-19 vaccine eligibility to include adults aged 65 years and older [20]. Following this expansion, vaccination uptake increased rapidly, with substantially higher coverage observed by 31 May 2021. In addition, vaccine supply expanded considerably beginning in March 2021, with distribution through retail pharmacy partners such as Kroger, Walmart, Walgreens, and independent pharmacies across Kentucky [21]. Access was further enhanced by programs offering free round-trip transportation to and from vaccination appointments [21].

### 4.2. Correlation Between Vaccination Rate and the SDOHs at the Beginning of Vaccination

In Quarter 1 of vaccination, there was a negative correlation between older adults’ vaccination and disability rates at the ZIP code level, as well as between older adults’ vaccination and poverty rates (Table 4). Since COVID-19 impacted the older adult population more than any other age group [22], especially in terms of mortality [23], it is of great concern that certain older adult populations in the county were found to be less vaccinated against COVID-19 in Quarter 1 than other older adult groups. Additionally, the fact that these lower vaccination rates were observed in county regions with more disabled and impoverished older adults compounds the concern of this finding, as disability and poverty status are shown to be among the leading indicators of adverse COVID-19 infection outcomes [24].

### 4.3. The Evolution of the Correlation Between Vaccination Rate and Disability and Poverty Rates

The correlation between vaccination and disability rates at the ZIP code level changed substantially over time, as both its magnitude and statistical significance decreased from Quarter 1 to Quarter 6 (Table 4). Moreover, the only statistically significant difference in regression slopes across SDOH factors was in disability rate between Quarter 1 and Quarter 6. Therefore, disability appeared to be an SDOH that could be managed during a vaccination campaign for older adults. Conversely, according to bivariate analyses, the ZIP-code-level poverty rate appeared to be a static correlate of the ZIP-code-level vaccination rate throughout the vaccination campaign, as it remained a statistically significant correlate of vaccination rates across all three quarters and there was no statistically significant difference in the regression slopes of the three quarters.

### 4.4. Regions with the Highest and Lowest Vaccination Rates

In Quarter 1, the Inner East region had a vastly different older adult vaccination rate (35.4%) than the West and Southwest regions (12.8% and 12.6%, respectively) (Table 2). The observed differences in vaccination rates may be explained by underlying SDOH. The Inner East region has a higher proportion of residents with graduate-level education and higher employment rates than the West region, where fewer than half as many residents hold graduate degrees and employment levels are lower [25]. In addition, the Inner East reports the highest median annual family income ($152,846.2) [25]. Higher educational attainment has been associated with greater influenza vaccination uptake [26], and employment is linked to lower vaccine hesitancy, likely due to improved health literacy status [27], better access to healthcare, and exposure to workplace health promotion initiatives. This study’s finding that the regional poverty rate was a static correlate of regional vaccination rates among older adults throughout the vaccination campaign is consistent with these pre-existing findings across all age groups, as educational attainment and employment are largely fixed among the older adult population. It was also observed that the Inner East had the lowest disability rate among older adults in the county (Table 3), which aligns with the region’s higher COVID-19 vaccination rates.

### 4.5. Vaccination Rate in the County Region with the Highest Poverty Rate

The Central region had the highest proportion of older adults living below the poverty line (Table 3), which corresponded with the moderately lower vaccine uptake observed throughout the quarters (Table 2). A prior study found that individuals who faced economic difficulties during the pandemic were markedly more likely to exhibit vaccine hesitancy, highlighting the association of financial instability with health-related behaviors [28]. Higher levels of income and education were associated with increased likelihood of accepting vaccination without hesitation [29].

### 4.6. Vaccination Rate in the County Region with the Highest Disability Rate

The West region had very low overall vaccination rates [25]. The West also had the lowest or second-lowest older adult vaccination rates among the county regions (in Quarter 1, when the West had the second-lowest rate, it was only 0.2% higher than the region with the lowest rate, the Southwest) (Table 2). Moreover, the West region had the highest older adult disability rate. Since the majority of older adult residents in the region were Black (81.0%), it stands to reason that a majority of the disabled older adults in this region were also Black. The Black community often has a structural mistrust of healthcare institutions [25], so reaching this population should be prioritized in a vaccination campaign. However, the vaccination campaign in the county struggled to reach Black, disabled older adults in the region of the county with the second-highest percentage of older adults living below the federal poverty line (Table 3). The causes of low vaccine uptake among this population, affected by multiple SDOH factors known to increase vulnerability to disease, should be better understood to improve future vaccination campaigns.

By Quarter 6, the two-dose vaccination rate among White older adults in the West region was 100%, whereas it was 68.3% among Black older adults (Table 2). While the White older adult population in the West is quite smaller than the region’s Black older adult population, the White population of 1268 individuals is large enough to make a vaccination percentage estimate of 100% noteworthy (Table 1). Contrastingly, in the West region, there were 86 Hispanic older adults, and by Quarter 6, only 28.3% of this population had received two doses of the COVID-19 vaccine. A future investigation should analyze whether a lower disability rate among White older adults in this region caused disparities in the COVID-19 vaccine uptake. Additionally, analyses should investigate whether Black older adults and Hispanic older adults have lower vaccination rates for different reasons.

A better understanding of which disabilities most affect older adults in the West region will help address low vaccination rates among this population. Certain disabilities may be associated more negatively with vaccine uptake among older adults in the region than others, so specifically reaching out to these individuals during future vaccination campaigns would be an effective way to address the vaccination disparity within the county.

### 4.7. Vaccination Rate and the Share of Households with an Older Adult

The cause of the positive correlation between the share of households with a 65+ person in a ZIP code and vaccination rate should also be further investigated. It would be helpful for future population estimates to include whether these households had only people aged 65 or older or were intergenerational. This would provide greater insight into whether living in an intergenerational household is an indicator of an older adult being more likely to be vaccinated. This would aid in understanding why the Hispanic vaccination rate was so low throughout the study period (Table 2), as Hispanic people live in intergenerational households, in certain circumstances [30]. If most Hispanic older adults in Jefferson County live in intergenerational households, it would indicate that, within the Hispanic community, living in an intergenerational household does not increase older adults’ likelihood of vaccination.

### 4.8. Vaccination Rate Among Hispanic Older Adults

It is also important to note that among U.S. Hispanic older adults, language preference has been associated with lower influenza vaccination rates, highlighting a potential health disparity [30]. Trust in vaccine information was higher among college-aged Hispanic women when the message was delivered by a healthcare provider of the same race or ethnicity [31]. Increasing the visibility and representation of Hispanic healthcare professionals in research communication and media outreach may help build greater vaccine confidence within Hispanic communities [32].

### 4.9. Vaccination Rate Among Asian Older Adults

Furthermore, investigating whether Asian older adults in the county were more likely to live in an intergenerational household would provide further insight into whether living in such households increases vaccination rates among older adults outside the Hispanic community. Although the county’s Asian older adult population was small, they were vaccinated at a very high rate. The Asian population is extremely diverse, but in general, the high vaccination rates seen among Asians are often attributed to higher education and income levels. However, living in intergenerational households may be another contributing factor to high vaccination rates among this population.

The higher vaccination rates observed among Asian older adults might also be attributable to lower disability prevalence among Asians in this age group compared to other racial and ethnic groups of the same age group [33]. Hence, an important future analysis is to investigate whether Asians are disabled at a lower rate than Hispanics, Multiracial people, Black people, or people of Some Other Races in the county. If they are not disabled at a lower rate, their higher COVID-19 vaccination rate may be due to income or education differences.

### 4.10. Other ACS ZIP-Code-Level Variables

While the ACS includes population estimates of grandparents living with grandchildren and other similar SDOH factors, it does not provide information on whether these grandparents are 65 or older. Additionally, the ACS does not provide racial and ethnic categorization of individuals within these SDOH factors, nor does it provide disability rates. Collecting SDOH information with greater granularity would enable public health practitioners to make far more informed decisions during future vaccination campaigns.

### 4.11. Limitations

This research has several limitations that should be considered when interpreting the findings. First, the vaccination rate estimates were based on population estimates that did fall within the margin of error of the American Community Survey (ACS), but they were closer to the low end of many ACS margins of error. Therefore, the analysis likely lacks some robustness against estimation error. This issue also led to a slight decrease in the cumulative vaccination rate from Quarter 2 to Quarter 6. Second, the analyzed SDOHs lacked race breakdowns. Therefore, when analyzing the issues the vaccination campaign faced in the West region, it was logically assumed that many of the region’s vaccinated older adults were Black and disabled, but it cannot be said with certainty how many of these older adults fit into either category. Third, while identifying disability as a correlate of COVID-19 vaccine disparity among older adults is critical for the development of tailored public health interventions, the ACS data did not provide sufficient granularity to identify the underlying mechanisms (e.g., cognitive health status, the presence of home-bound conditions, or the specific influence of various Medicare coverage types on access to vaccination sites) behind the correlation. Fourth, the results for the association of vaccination and poverty rates should be interpreted with caution as they were not statistically significant at the 5% level in the multivariate analyses. Larger sample sizes are needed to investigate the association further. Fifth, the ecological nature of the presented spatial analysis means that ZIP code-level associations between socioeconomic factors and vaccine uptake describe community-level trends rather than individual-level behaviors—a distinction particularly important for older adults whose mobility and access to technology may vary significantly within a single neighborhood. Sixth, the reliance on KYIR data introduces the possibility of administrative undercounting; for instance, older adults who received vaccinations through federal programs (such as the Veterans Health Administration) or out-of-state providers might not be fully captured in the local registry. Seventh, as a single-site study restricted to Jefferson County, Kentucky, the results may not be fully generalizable to older adult populations in different geographic or socio-political contexts, particularly those in rural areas or states with different public health infrastructures.

## 5. Conclusions

This study demonstrated that while overall COVID-19 vaccination coverage among older adults in Jefferson County, Kentucky, increased significantly over time, deep-seated geographical and sociodemographic disparities persisted. The spatial analysis revealed that even within a single county, neighborhood-level environments—characterized by varying levels of disability and poverty—could play a role in predisposing older adults to, or enabling them to, take up vaccines. While the gap between high-uptake county regions like the Inner East and lower-uptake county regions like the West narrowed by the sixth quarter, the structural inequities driving these initial delays remained a public health challenge.

A finding of this research was the inverse correlation between ZIP-code-level disability rate and vaccination coverage among those aged 65 and older. The inverse correlation between disability rate and vaccination coverage in the early stages of the vaccination campaign suggests that conventional outreach strategies may be insufficient for older adults with functional impairments. Policies, hence, should focus on hyper-local, ZIP code-level strategies that directly address the specific needs of disabled and socioeconomically disadvantaged older adults, ensuring that geography and physical ability do not dictate access to life-saving preventive care.

## Figures and Tables

**Table 1:** Jefferson County, Kentucky, older adult population in 2021 by county region, race, and ethnicity.

		Race						
						Some	Ethnicities	
County					Multi-	Other		Non-
Region	Overall	White	Black	Asian	racial	Races	Hispanic	Hispanic
Frequencies:								
Overall	139,222	111,061	20,864	2362	4417	1145	3103	136,422
West	7888	1268	6388	15	176	40	86	7821
Southwest	27,227	21,458	4183	495	1080	224	703	26,602
South	30,846	24,422	4295	574	1333	478	1140	29,800
Central	8193	5777	2082	74	223	56	147	8076
Inner East	15,686	14,612	575	140	332	56	170	15,534
Outer East	48,630	42,819	3319	1058	1274	292	852	47,844
Percentages:								
	Vertical %s	Horizontal %s						
Overall	100	79.8	15.0	1.7	3.2	0.8	2.2	98.0
West	5.7	16.1	81.0	0.2	2.2	0.5	1.1	98.9
Southwest	19.6	78.8	15.4	1.8	4.0	0.8	2.6	97.4
South	22.2	79.2	13.9	1.9	4.3	1.5	3.7	96.3
Central	5.9	70.5	25.4	0.9	2.7	0.7	1.8	98.2
Inner East	11.3	93.2	3.7	0.9	2.1	0.4	1.1	98.9
Outer East	34.9	88.1	6.8	2.2	2.6	0.6	1.7	98.3

*Source:* Authors’ estimates using ZIP code-level population data by race from the Censuses 2010 and 2020. *Notes:* Two races (American Indians and Alaska Natives, and Native Hawaiian or Other Pacific Islanders) are not included in the population estimates due to their small numbers in the county.

**Table 2:** Two-dose COVID-19 vaccination rate among older adults at the end of the first, second, and sixth quarters of the vaccination campaign in Jefferson County, Kentucky, on 28 February 2021, 31 May 2021, and 31 May 2022, respectively, by county region, race, and ethnicity.

			Races						
							Some	Ethnicities	
Assessment	County					Multi-	Other	Non-	
Date	Region	Overall	White	Black	Asian	racial	Races	Hispanic	Hispanics
First Quarter (Q1) 2/28/2021	Overall	22.4	23.7	16.5	18.4	19.0	17.9	22.7	8.0
	West	12.8	17.2	11.9	52.0	12.5	5.1	12.9	2.3
	Southwest	12.6	13.0	10.7	8.9	12.0	12.0	12.8	5.5
	South	15.4	15.9	12.8	7.7	15.3	13.2	15.7	6.7
	Central	21.2	22.2	15.5	53.2	24.7	17.6	21.4	8.8
	Inner East	35.4	34.9	50.6	35.5	31.3	25.3	35.6	14.1
	Outer East	29.8	29.7	32.9	24.0	25.0	30.2	30.1	11.3
Second Quarter (Q2) 5/31/2021	Overall	70.5	71.6	65.5	67.8	65.9	48.9	71.0	47.4
	West	62.9	85.4	58.5	100.0	60.7	20.2	63.3	24.4
	Southwest	64.4	65.2	58.3	66.3	64.0	53.6	64.7	51.1
	South	67.4	68.2	63.0	58.0	65.2	45.7	68.0	48.2
	Central	67.4	70.0	57.3	95.7	61.9	43.9	67.8	41.9
	Inner East	78.0	76.6	100.0	98.1	67.4	43.4	78.4	43.6
	Outer East	75.7	74.9	90.1	67.3	68.8	56.3	76.2	48.9
Sixth Quarter (Q6) 5/31/2022	Overall	77.5	78.3	73.3	73.9	64.6	51.4	77.9	58.1
	West	74.1	100.0	68.3	100.0	70.5	21.0	74.6	28.3
	Southwest	74.1	75.5	63.6	74.8	63.5	61.1	74.2	66.7
	South	75.5	76.6	68.9	61.9	64.8	48.9	75.9	60.6
	Central	76.7	78.6	70.1	100.0	64.0	46.2	77.1	51.1
	Inner East	83.3	81.5	100.0	100.0	66.3	38.2	83.6	56.3
	Outer East	80.0	79.0	95.3	73.4	63.9	56.3	80.4	53.6
Change from Q1 to Q2	Overall	48.1	47.9	49.0	49.4	46.9	31.0	48.3	39.4
	West	50.1	68.2	46.6	48.0	48.2	15.1	50.4	22.1
	Southwest	51.8	52.2	47.6	57.4	52.0	41.6	51.9	45.6
	South	52.0	52.3	50.2	50.3	49.9	32.5	52.3	41.5
	Central	46.2	47.8	41.8	42.5	37.2	26.3	46.4	33.1
	Inner East	42.6	41.7	49.4	62.6	36.1	18.1	42.8	29.5
	Outer East	45.9	45.2	57.2	43.3	43.8	26.1	46.1	37.6
Change from Q2 to Q6	Overall	7.0	6.7	7.8	6.1	0.0	2.5	6.9	10.7
	West	11.2	14.6	9.8	0.0	9.8	0.8	11.3	3.9
	Southwest	9.7	10.3	5.3	8.5	0.0	7.5	9.5	15.6
	South	8.1	8.4	5.9	3.9	0.0	3.2	7.9	12.4
	Central	9.3	8.6	12.8	4.3	2.1	2.3	9.3	9.2
	Inner East	5.3	4.9	0.0	1.9	0.0	0.0	5.2	12.7
	Outer East	4.3	4.1	5.2	6.1	0.0	0.0	4.2	4.7

*Notes:* The population of Asian older adults in the West and Central regions, the population of older adults of Some Other Races in the West, Central, and Inner East Regions, and the population of Hispanic older adults in the West region were estimated to be fewer than 100 in 2021. Therefore, the estimated vaccination rates for these populations should be interpreted with caution. The green colors follow the pattern in Figure 1, Figure 2 and Figure 3: the higher the vaccination rate, the darker the green. The orange color pattern shows the extent of the increase in vaccination rates from Q1 to Q2 and from Q2 to Q6: the greater the increase, the darker the orange.

**Table 3:** Older adult population and SDOH estimates for Jefferson County, Kentucky, regions in 2021.

County Region	Percentage of Older Adults Living with a Disability	Percentage of Older Adults Living Below Poverty Line	Percentage of Households with an Older Adult	Percentage of Older Adult Householders Living Alone
Overall	34.7	10.0	29.8	49.0
West	51.4	19.7	28.6	56.4
Southwest	40.5	13.4	28.8	48.8
South	35.2	9.3	28.0	47.1
Central	41.8	26.0	21.4	67.9
Inner East	26.3	5.6	31.1	51.9
Outer East	29.8	5.4	33.1	44.0

**Table 4:** ZIP-code-level correlation of vaccination rate and SDOH at the end of Quarters 1, 2, and 3 of the COVID-19 vaccination campaign in Jefferson County, Kentucky (N = 34).

Quarter of Vaccination Rate Assessment	SDOH Indicators:			
	Disability Rate	Poverty Rate	The Share of Households with a 65+ Person	The Rate of 65+ Householders Living Alone
Pearson Correlation Coefficient:				
Quarter 1	−0.48	−0.41	0.39	−0.02
Quarter 2	−0.28	−0.51	0.48	−0.04
Quarter 6	−0.07	−0.36	0.35	0.17
The Slope of the Regression of Vaccination Rate on the SDOH:				
Quarter 1	−0.38	−0.36	0.7	−0.01
	(−0.63, −0.13) *	(−0.65, −0.07) *	(0.10, 1.30) *	(−0.27, 0.24)
Quarter 2	−0.2	−0.41	0.79	−0.03
	(−0.45, 0.05)	(−0.66, −0.16) *	(0.27, 1.31) *	(−0.26, 0.21)
Quarter 6	−0.04	−0.25	0.5	0.1
	(−0.27, 0.18)	(−0.49, −0.02) *	(0.01, 0.99) *	(−0.10, 0.31)

*Notes:* The denominator for the rate of 65+ householders living alone is the total number of 65+ householders. Numbers in parentheses are 95 percent confidence intervals. The asterisk indicates statistical significance at the 5% level.

## Data Availability

The datasets presented in this article are not readily available because the data set contains protected health information subject to HIPAA. Requests to access the datasets should be directed to the Kentucky Department of Public Health.

## Appendix A

**Table A1:** Older adult 2021 population in Jefferson County, Kentucky, by race, ethnicity, county region, and ZIP code.

			Races						
	County						Some	Ethnicities	
County	Region/					Multi-	Other	Non-	
Region	ZIP Code	Total	White	Black	Asian	racial	Races	Hispanic	Hispanic
Overall		139,222	111,061	20,864	2362	4417	1145	136,422	3103
County Regions	West	1268	6388	1268	15	176	40	7821	86
	Southwest	21,458	4183	21,458	495	1080	224	26,602	703
	South	24,422	4295	24,422	574	1333	478	29,800	1140
	Central	5777	2082	5777	74	223	56	8076	147
	Inner East	14,612	575	14,612	140	332	56	15,534	170
	Outer East	42,819	3319	42,819	1058	1274	292	47,844	852
West	40210	208	1849	208	7	65	13	2111	33
	40211	206	2918	206	3	46	27	3170	27
	40212	854	1621	854	5	65	0	2540	26
Southwest	40177	270	0	270	0	7	0	279	1
	40214	5198	638	5198	318	346	91	6264	283
	40215	1786	569	1786	35	134	28	2455	81
	40216	4591	2282	4591	69	262	48	7028	173
	40258	3619	401	3619	27	122	26	4113	66
	40272	5994	293	5994	46	209	31	6463	99
South	40118	1320	44	1320	8	49	27	1393	51
	40213	2006	335	2006	28	118	36	2423	95
	40218	2552	1716	2552	201	227	93	4540	210
	40219	4546	1027	4546	99	368	148	5730	389
	40228	2558	358	2558	72	141	33	3041	112
	40229	4716	250	4716	56	225	71	5154	144
	40291	6724	565	6724	110	205	70	7519	139
Central	40202	326	350	326	25	21	14	710	24
	40203	1034	1185	1034	18	65	18	2272	50
	40204	1971	151	1971	10	60	7	2172	29
	40208	773	299	773	7	32	16	1105	27
	40217	1673	97	1673	14	45	1	1817	17
Inner East	40205	5079	143	5079	39	92	17	5325	41
	40206	3002	274	3002	42	96	23	3382	56
	40207	6531	158	6531	59	144	16	6827	73
Outer East	40023	840	13	840	9	24	0	882	0
	40059	3900	186	3900	105	94	13	4244	51
	40220	5758	794	5758	139	153	52	6779	109
	40222	4360	181	4360	95	189	23	4698	136
	40223	4479	317	4479	82	102	34	4948	65
	40241	6148	478	6148	199	161	26	6910	99
	40242	1999	156	1999	33	73	15	2217	54
	40243	2549	133	2549	45	44	23	2746	39
	40245	5819	485	5819	212	177	45	6613	131
	40299	6967	576	6967	139	257	61	7807	168

**Table A2:** Older adult 2021 population shares in Jefferson County, Kentucky, by race, ethnicity, county region, and ZIP code.

			Horizontal Percentages						
			Races						
	County	Vertical					Some	Ethnicities	
County	Region/	Percent-				Multi-	Other	Non-	
Region	ZIP Code	ages	White	Black	Asian	racial	Races	Hispanic	Hispanic
Overall		100	79.8	15.0	1.7	3.2	0.8	98.0	2.2
County Regions	West	16.1	81.0	16.1	0.2	2.2	0.5	99.2	1.1
	Southwest	78.8	15.4	78.8	1.8	4.0	0.8	97.7	2.6
	South	79.2	13.9	79.2	1.9	4.3	1.5	96.6	3.7
	Central	70.5	25.4	70.5	0.9	2.7	0.7	98.6	1.8
	Inner East	93.2	3.7	93.2	0.9	2.1	0.4	99.0	1.1
	Outer East	88.1	6.8	88.1	2.2	2.6	0.6	98.4	1.8
West	40210	9.7	86.4	9.7	0.3	3.0	0.6	98.6	1.5
	40211	6.5	91.5	6.5	0.1	1.4	0.8	99.4	0.8
	40212	33.4	63.4	33.4	0.2	2.5	0.0	99.3	1.0
Southwest	40177	96.8	0.0	96.8	0.0	2.5	0.0	100.0	0.4
	40214	79.7	9.8	79.7	4.9	5.3	1.4	96.0	4.3
	40215	70.7	22.5	70.7	1.4	5.3	1.1	97.2	3.2
	40216	63.9	31.8	63.9	1.0	3.6	0.7	97.8	2.4
	40258	86.8	9.6	86.8	0.6	2.9	0.6	98.7	1.6
	40272	91.5	4.5	91.5	0.7	3.2	0.5	98.7	1.5
South	40118	91.7	3.1	91.7	0.6	3.4	1.9	96.7	3.5
	40213	80.0	13.4	80.0	1.1	4.7	1.4	96.6	3.8
	40218	53.9	36.3	53.9	4.2	4.8	2.0	95.9	4.4
	40219	74.6	16.9	74.6	1.6	6.0	2.4	94.0	6.4
	40228	81.5	11.4	81.5	2.3	4.5	1.1	96.9	3.6
	40229	89.2	4.7	89.2	1.1	4.3	1.3	97.5	2.7
	40291	87.9	7.4	87.9	1.4	2.7	0.9	98.3	1.8
Central	40202	44.5	47.8	44.5	3.4	2.9	1.9	97.0	3.3
	40203	44.7	51.2	44.7	0.8	2.8	0.8	98.2	2.2
	40204	89.9	6.9	89.9	0.5	2.7	0.3	99.0	1.3
	40208	68.7	26.6	68.7	0.6	2.8	1.4	98.2	2.4
	40217	91.5	5.3	91.5	0.8	2.5	0.1	99.3	0.9
Inner East	40205	94.7	2.7	94.7	0.7	1.7	0.3	99.3	0.8
	40206	87.5	8.0	87.5	1.2	2.8	0.7	98.6	1.6
	40207	94.7	2.3	94.7	0.9	2.1	0.2	99.0	1.1
Outer East	40023	94.2	1.5	94.2	1.0	2.7	0.0	98.9	0.0
	40059	90.9	4.3	90.9	2.4	2.2	0.3	98.9	1.2
	40220	83.8	11.6	83.8	2.0	2.2	0.8	98.6	1.6
	40222	90.3	3.8	90.3	2.0	3.9	0.5	97.3	2.8
	40223	89.5	6.3	89.5	1.6	2.0	0.7	98.8	1.3
	40241	87.8	6.8	87.8	2.8	2.3	0.4	98.7	1.4
	40242	88.1	6.9	88.1	1.5	3.2	0.7	97.7	2.4
	40243	91.6	4.8	91.6	1.6	1.6	0.8	98.7	1.4
	40245	86.5	7.2	86.5	3.2	2.6	0.7	98.3	1.9
	40299	87.5	7.2	87.5	1.7	3.2	0.8	98.1	2.1

**Table A3:** Two-dose COVID-19 vaccination rate among older adults at the end of the first quarter of the vaccination campaign on 28 February 2021 by race, ethnicity, county region, and ZIP code.

			Races						
County Region	County Region/ ZIP Code	Overall	White	Black	Asian	Multi- Racial	Some Other Races	Ethnicities	
								Non- Hispanic	Hispanic
Overall		22.4	23.7	16.5	18.4	19.0	17.9	22.7	8.0
County Regions	West	12.8	17.2	11.9	52.0	12.5	5.1	12.9	2.3
	Southwest	12.6	13.0	10.7	8.9	12.0	12.0	12.8	5.5
	South	15.4	15.9	12.8	7.7	15.3	13.2	15.7	6.7
	Central	21.2	22.2	15.5	53.2	24.7	17.6	21.4	8.8
	Inner East	35.4	34.9	50.6	35.5	31.3	25.3	35.6	14.1
	Outer East	29.8	29.7	32.9	24.0	25.0	30.2	30.1	11.3
West	40210	12.0	14.4	11.8	0.0	10.7	15.9	12.1	3.0
	40211	14.7	42.7	12.7	100.0	8.7	0.0	14.8	3.7
	40212	11.1	11.7	10.6	57.0	17.1	0.0	11.3	0.0
Southwest	40177	3.2	3.0	0.0	0.0	0.0	0.0	3.2	0.0
	40214	13.6	15.0	7.1	4.4	12.4	7.7	14.0	3.9
	40215	9.7	11.0	6.7	5.8	5.2	10.7	9.7	8.6
	40216	14.6	16.3	11.1	17.3	11.5	14.7	14.8	5.8
	40258	11.8	11.4	13.9	22.1	11.5	7.7	11.8	7.5
	40272	11.6	10.8	18.4	21.8	17.2	25.5	11.6	6.0
South	40118	7.8	7.7	11.3	0.0	6.1	7.4	7.7	9.9
	40213	16.3	17.7	10.5	0.0	15.2	5.6	16.6	8.4
	40218	14.9	17.3	11.9	4.5	15.9	16.2	15.4	4.3
	40219	15.3	16.2	10.7	12.2	14.7	17.5	16.0	4.9
	40228	16.3	17.1	14.2	6.9	13.5	6.0	16.5	10.7
	40229	11.3	11.3	10.8	3.6	13.8	4.3	11.3	9.0
	40291	19.3	19.2	20.9	14.6	21.0	18.6	19.5	7.2
Central	40202	17.7	26.4	10.6	4.0	19.1	14.5	18.0	8.4
	40203	21.5	28.2	13.7	68.1	38.7	34.1	21.8	6.1
	40204	27.4	27.9	16.6	69.3	31.6	29.9	27.7	6.8
	40208	14.9	18.0	8.4	26.8	6.3	0.0	14.9	11.2
	40217	20.3	19.1	35.0	76.0	20.1	0.0	20.4	11.5
Inner East	40205	35.1	34.4	64.0	35.6	30.4	6.0	35.2	24.6
	40206	30.0	30.3	28.5	23.5	25.0	39.0	30.4	7.2
	40207	38.3	37.3	77.1	44.1	36.0	25.5	38.6	13.6
Outer East	40023	17.6	17.4	29.9	10.7	25.2	0.0	17.7	0.0
	40059	37.1	36.8	53.2	31.5	19.2	55.9	37.4	17.8
	40220	25.7	26.7	18.9	18.0	26.2	32.8	25.9	11.9
	40222	36.0	35.0	60.8	31.6	35.4	34.8	36.7	13.2
	40223	30.9	30.4	40.1	24.4	26.5	35.2	31.2	6.2
	40241	35.3	35.4	36.6	31.1	31.0	30.9	35.6	13.2
	40242	28.9	29.5	27.6	27.5	16.5	26.6	29.5	5.6
	40243	30.1	28.2	64.1	44.9	18.0	22.2	30.4	7.7
	40245	27.7	28.0	30.5	17.0	20.9	22.3	28.0	9.9
	40299	23.2	23.1	26.4	12.9	21.0	27.6	23.5	10.7

*Notes:* The population of Asian older adults in the West and Central regions, the population of older adults of Some Other Races in the West, Central, and Inner East Regions, and the population of Hispanic older adults in the West region were estimated to be fewer than 100 in 2021. Therefore, the estimated vaccination rates for these populations should be interpreted with caution. The green colors follow the pattern in Figure 1, Figure 2 and Figure 3: the higher the vaccination rate, the darker the green.

**Table A4:** Two-dose COVID-19 vaccination rate among older adults at the end of the second quarter of the vaccination campaign on 31 May 2021 by race, ethnicity, county region, and ZIP code.

			Races						
County Region	County Region/ ZIP Code	Overall	White	Black	Asian	Multi- Racial	Some Other Races	Ethnicities	
								Non- Hispanic	Hispanic
Overall		70.5	71.6	65.5	67.8	65.9	48.9	71.0	47.4
County Regions	West	62.9	85.4	58.5	100.0	60.7	20.2	63.3	24.4
	Southwest	64.4	65.2	58.3	66.3	64.0	53.6	64.7	51.1
	South	67.4	68.2	63.0	58.0	65.2	45.7	68.0	48.2
	Central	67.4	70.0	57.3	95.7	61.9	43.9	67.8	41.9
	Inner East	78.0	76.6	100.0	98.1	67.4	43.4	78.4	43.6
	Outer East	75.7	74.9	90.1	67.3	68.8	56.3	76.2	48.9
West	40210	63.2	100.0	59.3	100.0	52.0	23.8	63.8	23.9
	40211	66.5	100.0	60.3	100.0	47.6	18.5	66.9	14.9
	40212	58.0	64.2	54.2	100.0	79.1	0.0	58.2	34.7
Southwest	40177	40.4	38.9	0.0	0.0	44.8	0.0	40.2	100.0
	40214	66.5	69.3	44.2	57.0	65.5	55.0	67.0	53.0
	40215	57.6	59.5	45.9	89.4	64.8	57.1	57.9	47.9
	40216	66.6	68.5	61.0	79.4	65.8	50.4	66.9	50.3
	40258	67.3	66.4	71.0	100.0	63.1	46.0	67.4	58.7
	40272	61.9	61.2	73.5	65.5	59.7	57.4	62.1	44.4
South	40118	61.5	61.7	56.3	83.5	51.2	55.9	61.9	47.5
	40213	69.6	72.3	58.0	43.5	61.0	50.1	70.2	51.6
	40218	68.5	73.9	61.8	48.2	65.7	58.3	69.8	37.6
	40219	64.7	66.2	56.0	63.9	63.9	45.2	65.9	45.2
	40228	71.7	73.0	67.0	55.4	63.3	50.9	71.6	71.6
	40229	59.8	59.1	67.6	65.9	69.3	14.2	60.0	49.3
	40291	72.6	72.2	78.4	70.1	69.8	53.0	73.0	50.9
Central	40202	59.4	66.5	55.7	12.1	71.4	29.0	60.3	33.7
	40203	59.8	69.2	50.1	100.0	74.4	68.1	60.5	26.3
	40204	69.6	70.4	57.0	100.0	64.9	74.7	70.2	23.9
	40208	55.0	56.5	52.1	67.0	59.5	25.2	55.8	22.5
	40217	73.4	72.3	93.7	100.0	53.6	0.0	73.3	80.6
Inner East	40205	79.2	77.2	100.0	100.0	69.6	29.8	79.4	59.1
	40206	72.0	73.2	66.1	56.5	61.3	57.0	72.6	35.9
	40207	80.1	77.7	100.0	100.0	70.0	38.3	80.5	40.9
Outer East	40023	65.6	65.0	100.0	53.3	58.8	0.0	65.6	0.0
	40059	74.4	73.1	100.0	66.9	54.3	63.9	74.7	55.4
	40220	75.9	77.3	67.4	56.3	78.5	67.6	76.2	53.3
	40222	76.3	74.1	100.0	72.7	72.4	52.2	77.5	33.7
	40223	80.0	78.6	100.0	80.4	64.9	67.5	80.4	50.8
	40241	77.5	76.3	98.1	70.8	68.8	50.2	77.8	54.7
	40242	74.9	75.3	73.1	73.5	64.8	73.1	75.8	37.2
	40243	80.4	78.0	100.0	100.0	69.8	31.0	80.6	64.2
	40245	70.5	69.8	87.4	60.5	65.4	44.7	71.1	41.9
	40299	75.8	75.4	84.2	59.6	71.3	56.9	76.3	53.5

*Notes:* The population of Asian older adults in the West and Central regions, the population of older adults of Some Other Races in the West, Central, and Inner East Regions, and the population of Hispanic older adults in the West region were estimated to be fewer than 100 in 2021. Therefore, the estimated vaccination rates for these populations should be interpreted with caution. The green colors follow the pattern in Figure 1, Figure 2 and Figure 3: the higher the vaccination rate, the darker the green.

**Table A5:** Two-dose COVID-19 vaccination rate among older adults at the end of the sixth quarter of the vaccination campaign on 31 May 2022 by race, ethnicity, county region, and ZIP code.

			Races						
County Region	County Region/ ZIP Code	Overall	White	Black	Asian	Multi- Racial	Some Other Races	Ethnicities	
								Non- Hispanic	Hispanic
Overall		77.5	78.3	73.3	73.9	64.6	51.4	77.9	58.1
County Regions	West	74.1	100.0	68.3	100.0	70.5	21.0	74.6	28.3
	Southwest	74.1	75.5	63.6	74.8	63.5	61.1	74.2	66.7
	South	75.5	76.6	68.9	61.9	64.8	48.9	75.9	60.6
	Central	76.7	78.6	70.1	100.0	64.0	46.2	77.1	51.1
	Inner East	83.3	81.5	100.0	100.0	66.3	38.2	83.6	56.3
	Outer East	80.0	79.0	95.3	73.4	63.9	56.3	80.4	53.6
West	40210	75.1	100.0	69.6	100.0	68.9	31.5	75.9	26.8
	40211	78.7	100.0	70.8	100.0	66.0	16.5	79.2	13.4
	40212	67.8	78.0	62.5	100.0	75.2	0.0	68.0	46.3
Southwest	40177	46.6	45.5	0.0	0.0	40.1	0.0	46.4	100.0
	40214	76.3	80.0	50.0	62.3	62.1	61.4	76.5	69.8
	40215	68.7	70.7	55.8	100.0	64.7	54.1	68.8	63.4
	40216	75.3	78.8	65.2	92.0	65.6	63.0	75.4	67.0
	40258	78.0	77.5	75.3	100.0	61.6	58.2	78.1	65.5
	40272	71.6	70.7	79.8	88.1	64.6	65.8	71.7	61.3
South	40118	71.9	71.6	63.9	91.0	66.7	54.9	72.0	65.1
	40213	80.4	83.2	67.9	53.7	65.3	59.4	80.5	71.6
	40218	77.2	84.4	68.1	50.1	66.1	59.1	78.5	45.9
	40219	75.1	77.2	61.8	67.5	63.1	52.1	76.0	58.9
	40228	79.5	81.3	71.3	56.4	63.4	57.1	79.0	88.9
	40229	69.1	68.8	76.2	70.8	67.5	14.4	69.3	59.4
	40291	76.5	76.1	80.6	78.7	64.1	58.6	76.9	57.1
Central	40202	69.4	77.2	65.6	13.7	73.3	37.9	70.0	49.1
	40203	71.4	81.1	62.1	96.6	73.6	62.8	72.2	31.9
	40204	76.1	76.7	67.9	100.0	59.6	66.9	76.7	33.4
	40208	64.5	65.5	61.7	86.2	73.9	33.4	65.0	42.1
	40217	82.8	80.9	100.0	100.0	57.9	0.0	82.7	84.8
Inner East	40205	83.8	81.4	100.0	100.0	68.2	23.0	83.8	87.5
	40206	78.7	79.1	79.8	64.3	68.2	55.0	79.3	41.9
	40207	85.3	82.7	100.0	100.0	64.0	31.7	85.7	51.0
Outer East	40023	68.7	67.3	100.0	82.0	63.7	0.0	68.5	0.0
	40059	76.3	74.2	100.0	75.2	57.1	63.1	76.5	56.3
	40220	81.7	83.4	72.6	60.7	72.9	63.7	82.0	61.1
	40222	83.3	80.7	100.0	85.7	65.8	60.6	84.7	35.9
	40223	85.6	83.9	100.0	89.0	66.5	72.0	86.0	58.8
	40241	81.1	79.8	100.0	79.5	63.4	54.8	81.3	65.3
	40242	81.2	81.5	83.0	74.9	60.4	74.6	82.2	41.4
	40243	85.9	83.6	100.0	100.0	62.0	30.0	86.1	69.2
	40245	71.7	70.8	89.2	66.0	63.2	40.2	72.3	41.8
	40299	80.2	79.9	88.6	58.2	61.3	58.9	80.6	57.1

*Notes:* The population of Asian older adults in the West and Central regions, the population of older adults of Some Other Races in the West, Central, and Inner East Regions, and the population of Hispanic older adults in the West region were estimated to be fewer than 100 in 2021. Therefore, the estimated vaccination rates for these populations should be interpreted with caution. The green colors follow the pattern in Figure 1, Figure 2 and Figure 3: the higher the vaccination rate, the darker the green.

**Table A6:** Older adult population and SDOH estimates for Jefferson County, Kentucky, regions and ZIP codes in 2021.

County Region	County Region/ ZIP Code	Percentage of Older Adults Living with a Disability	Percentage of Older Adults Living Below Poverty Line	Percentage of Households with an Older Adult	Percentage of Older Adult Householders Living Alone
Overall		34.7	10.0	29.8	49.0
County Regions	West	51.4	19.7	28.6	56.4
	Southwest	40.5	13.4	28.8	48.8
	South	35.2	9.3	28.0	47.1
	Central	41.8	26.0	21.4	67.9
	Inner East	26.3	5.6	31.1	51.9
	Outer East	29.8	5.4	33.1	44.0
West	40210	55.0	24.4	29.2	64.7
	40211	48.3	16.6	25.7	52.4
	40212	52.7	20.1	31.5	54.4
Southwest	40177	31.5	20.2	30.6	30.6
	40214	39.8	13.0	24.9	45.8
	40215	59.1	17.1	23.2	55.9
	40216	39.6	18.9	32.2	57.4
	40258	32.7	12.5	26.2	46.6
	40272	41.7	6.9	32.4	40.6
South	40118	66.6	14.9	32.2	49.1
	40213	34.7	8.4	30.0	56.0
	40218	33.6	10.4	23.3	55.3
	40219	33.7	12.5	26.7	48.6
	40228	33.9	15.3	27.3	47.8
	40229	39.9	5.8	24.1	38.3
	40291	29.1	5.5	32.5	42.6
Central	40202	22.2	54.9	20.9	89.8
	40203	51.4	38.7	20.0	82.3
	40204	28.9	9.1	19.4	53.2
	40208	31.9	12.3	18.3	48.9
	40217	56.4	24.7	26.9	68.6
Inner East	40205	27.4	6.6	33.2	52.9
	40206	27.1	6.4	22.0	53.2
	40207	25.0	4.5	34.0	50.4
Outer East	40023	21.8	4.2	30.2	17.8
	40059	22.8	0.6	36.9	30.9
	40220	33.4	6.6	30.3	50.2
	40222	29.0	7.0	33.3	52.4
	40223	34.3	5.1	34.8	46.3
	40241	28.5	2.9	36.4	46.5
	40242	26.1	4.3	29.0	45.7
	40243	55.6	7.3	39.4	53.3
	40245	23.8	9.2	28.3	39.9
	40299	26.8	5.3	31.1	38.1

**Table A7:** ZIP-code-level correlation of vaccination rate and SDOH at the end of Quarters 1, 2, and 3 of the COVID-19 vaccination campaign in Jefferson County, Kentucky (ZIP codes within the top and bottom 10% of the SDOH were dropped) (N = 26).

Quarter of Vaccination Rate Assessment	SDOH Indicators:			
	Disability Rate	Poverty Rate	The Share of Households with a 65+ Person	The Rate of 65+ Householders Living Alone
Pearson Correlation Coefficient:				
Quarter 1	−0.56	−0.70	0.28	0.05
Quarter 2	−0.48	−0.70	0.19	0.05
Quarter 6	−0.29	−0.61	0.07	0.19
The Slope of the Regression of Vaccination Rate on the SDOH:				
Quarter 1	−0.63	−0.17	0.73	1.46
	(−1.02, −0.24) *	(−0.91, 0.57) *	(−0.33, 1.79)	(−0.47, 3.38)
Quarter 2	−0.50	−0.24	0.45	0.60
	(−0.90, −0.11) *	(−0.83, 0.34) *	(−0.54, 1.43) *	(−0.91, 2.12)
Quarter 6	−0.27	−0.20	0.14	0.42
	(−0.65, 0.11) *	(−0.67, 0.27) *	(−0.78, 1.07) *	(−0.72, 1.56) *

*Notes:* The denominator for the rate of 65+ householders living alone is the total number of 65+ householders. 95 percent confidence intervals are reported in parentheses. The asterisk indicates statistical significance at this level, 5%.

**Table A8:** Correlation Matrix of the Potential Predictors of Older Adults’ ZIP Code Level Vaccination Rates (N = 34).

	Disability Rate	Poverty Rate	The Share of Households with a 65+ Person	The Rate of 65+ Householders Living Alone
65+ Disability Rate	1.0000			
The Share of Households with a 65+ Person	0.3128	1.0000		
65+ Poverty Rate	−0.0186	−0.4744	1.0000	
Rate of 65+ Householders Living Alone	0.3497	0.7752	−0.3962	1.0000

**Table A9:** ZIP Code Level Association of Older Adults’ Vaccination and Disability Rates at the ZIP Code Level at the End of Quarters 1, 2, and 3 of COVID-19 Vaccination Campaign through Stepwise Addition of the Share of Households with an Older Adult and Poverty Rate (N = 34).

Quarter of Vaccination Rate Assessment	Coefficient of Disability Rate		
		Added:	Added:
		The Share of	65+
		Households with	Poverty
	No Covariates	a 65+ Person	Rate
Quarter 1	−0.38	−0.37	−0.35
	(−0.63, −0.13) *	(−0.60, −0.15) *	(−0.59, −0.10) *
Quarter 2	−0.20	−0.20	−0.13
	(−0.45, 0.05)	(−0.42, 0.02)	(−0.36, 0.10)
Quarter 6	−0.04	−0.04	0.01
	(−0.27, 0.18)	(−0.26, 0.18)	(−0.22, −0.24)

*Notes:* 95 percent confidence intervals are reported in parentheses. The asterisk indicates statistical significance at the 5% level.

**Table A10:** ZIP Code Level Association Older Adults’ Vaccination and Poverty Rates at the ZIP Code Level at the End of Quarters 1, 2, and 3 of COVID-19 Vaccination Campaign through Stepwise Addition of the Share of Households with an Older Adult and Disability Rate (N = 34).

Quarter of Vaccination Rate Assessment	Coefficient of Poverty Rate:		
		Added:	Added:
		The Share of	65+
		Households with	Disability
	No Covariates	a 65+ Person	Rate
Quarter 1	−0.36	−0.26	−0.10
	(−0.65, −0.07) *	(−0.58, 0.07)	(−0.42, 0.21)
Quarter 2	−0.41	−0.29	−0.24
	(−0.66, −0.16) *	(−0.57, −0.02) *	(−0.53, 0.05)
Quarter 6	−0.25	−0.18	−0.18
	(−0.49, −0.02) *	(−0.45, 0.09)	(−0.47, 0.11)

*Notes:* 95 percent confidence intervals are reported in parentheses. The asterisk indicates statistical significance at the 5% level.

**Table A11:** ZIP Code Level Association Older Adults’ Vaccination Rate and the Share Households with an Older Adult at the ZIP Code Level at the End of Quarters 1, 2, and 3 of COVID-19 Vaccination Campaign through Stepwise Addition of Disability and Poverty Rates (N = 34).

Quarter of Vaccination Rate Assessment	Coefficient of the Share Households with an Older Adult:		
		Added:	Added:
		65+	65+
		Disability	Poverty
	No Covariates	Rate	Rate
Quarter 1	0.70	0.68	0.58
	(0.10, 1.30) *	(0.16, 1.20) *	(−0.02, 1.19)
Quarter 2	0.79	0.78	0.56
	(0.27, 1.31) *	(0.28, 1.29) *	(−0.01, 1.12)
Quarter 6	0.50	0.50	0.32
	(0.01, 0.99) *	(0.00, 0.99) *	(−0.24, 0.89)

*Notes:* 95 percent confidence intervals are reported in parentheses. The asterisk indicates statistical significance at the 5% level.
